# Animal Models of Osteochondral Defect for Testing Biomaterials

**DOI:** 10.1155/2020/9659412

**Published:** 2020-01-28

**Authors:** Xiangbo Meng, Reihane Ziadlou, Sibylle Grad, Mauro Alini, Chunyi Wen, Yuxiao Lai, Ling Qin, Yanyan Zhao, Xinluan Wang

**Affiliations:** ^1^College of Pharmaceutical Sciences, Hebei University, Baoding, China; ^2^Translational Medicine R&D Center, Institute of Biomedical and Health Engineering, Shenzhen Institutes of Advanced Technology, Chinese Academy of Sciences, Shenzhen, China; ^3^AO Research Institute Davos, Clavadelerstrasse 8, 7270 Davos Platz, Switzerland; ^4^Department of Biomedical Engineering, Faculty of Engineering, The Hong Kong Polytechnic University, Hung Hom, Kowloon, Hong Kong SAR, China; ^5^Musculoskeletal Research Laboratory, Department of Orthopaedics & Traumatology, The Chinese University of Hong Kong, Hong Kong SAR, China

## Abstract

The treatment of osteochondral defects (OCD) remains a great challenge in orthopaedics. Tissue engineering holds a good promise for regeneration of OCD. In the light of tissue engineering, it is critical to establish an appropriate animal model to evaluate the degradability, biocompatibility, and interaction of implanted biomaterials with host bone/cartilage tissues for OCD repair *in vivo*. Currently, model animals that are commonly deployed to create osteochondral lesions range from rats, rabbits, dogs, pigs, goats, and sheep horses to nonhuman primates. It is essential to understand the advantages and disadvantages of each animal model in terms of the accuracy and effectiveness of the experiment. Therefore, this review aims to introduce the common animal models of OCD for testing biomaterials and to discuss their applications in translational research. In addition, we have reviewed surgical protocols for establishing OCD models and biomaterials that promote osteochondral regeneration. For small animals, the non-load-bearing region such as the groove of femoral condyle is commonly chosen for testing degradation, biocompatibility, and interaction of implanted biomaterials with host tissues. For large animals, closer to clinical application, the load-bearing region (medial femoral condyle) is chosen for testing the durability and healing outcome of biomaterials. This review provides an important reference for selecting a suitable animal model for the development of new strategies for osteochondral regeneration.

## 1. Introduction

Osteochondral defects (OCD) are a common condition caused by severe trauma, sports injuries, or physical diseases, leading to joint pain, deformity, and dysfunction [1]. Joint injuries caused by trauma and sports accidents often progress into osteoarthritis (OA). So, OCD are also a significant cause of OA [2]. OA has been reported to be the third most common musculoskeletal disease in the world [3]. The global prevalence of OA for persons older than 60 years is estimated at 33.6% for women and 24.3% for men [4]. As cartilage has no vasculature and lymphatic vessels and mature chondrocytes have limited proliferation and migration capabilities, cartilage regeneration remains a major challenge. OCD including lesions or degeneration of cartilage, subchondral bone, and bone-cartilage interfaces are notorious for being unable to heal. In order to repair OCD, the tissue complex of bone, cartilage, and bone-cartilage interfaces must be taken into account for repair and regeneration [5, 6]. Yet, those OCD are difficult to treat because the cartilage and the subchondral bone are tissues with different intrinsic healing capacities.

The current clinical treatments for repair of OCD are only palliative rather than curative [7]. The common goal of successful treatments is to relieve pain, repair damaged tissue, and improve joint function [8]. Current methods for treatment of cartilage lesions mainly include medical treatments (nonsteroid anti-inflammatory drugs (NSAIDs), pain killers, and hormones, etc.) and surgical treatment (arthroscopic lavage and debridement, cell-based therapy, and tissue-based therapy) [9]. Unfortunately, the medical treatments only relieve pain, rather than restoring the structural integrity of the articular cartilage [10], and the surgical treatments cannot restore neo-tissue close to normal cartilage [9]. Therefore, the treatment effect is not ideal, and the development of new treatment strategies is an urgent need. However, any new treatment strategy must be tested in animals to ensure its safety, feasibility, and effectiveness before clinical testing. It is very important to simulate human symptoms using appropriate animal models before clinical trials. At the same time, animal models are effective for developing OCD repair methods. Therefore, it is crucial to establish a suitable animal model for evaluating the effectiveness and safety of new treatment strategies.

In this review, we summarize the benefits and limitations of each species for reproducing specific defects, analyze and compare the similarities between animal models and human clinical conditions, and emphasize the factors that need to be considered when selecting animals.

## 2. Selection Criteria and Critical Size

### 2.1. General Selection Criteria

The ideal animal model should be as close to the clinical setting as possible, have biological similarity, and be a suitable model for cartilage physiology [11, 12]. A range of factors must be considered to select an applicable animal model for OCD regeneration. Before selecting an ideal animal model, it is crucial to decide whether a small or large animal model would be suitable for a particular OCD regeneration. The small animal models for OCD regeneration include rats and rabbits [13], while large animal models for OCD repair include dogs, pigs, sheep, goats, and horses [14]. Every animal has its advantages and limitations. When assessing the clinical potential of new strategies, the animal model that most closely represents human anatomy and physiology should be selected [15]. In addition, when investigating articular osteochondral repair *in vivo*, the factors to be considered include joint size, cartilage thickness, defect depth and diameter, skeletal maturity age, joint load distribution, and affordability and convenience of animal handling (Table 1) [16–18].

### 2.2. Critical Size of OCD

The critical size defect is defined as the smallest defect size (in diameter) the animal cannot self-repair without intervention [19]. In animal experiments, the understanding of critical-sized defects is crucial for reducing costs and animal suffering, at the same time still providing reliable data on the research results. So, the critical size of the defect should be considered to select the appropriate animal model for OCD repair. Katagiri et al. found that, in the rat knee, OCD with a diameter of 1.4 mm and a depth of 1.0 mm could not spontaneously recover the osteochondral unit, thus defining the critical size of rat knee osteochondral injury [20], whereby the mean animal weight is about 0.3 kg. The critical-sized defect of the rabbit knee has been defined as 3 mm, which can prevent spontaneous healing [21]. This dimension has, however, been questioned due to reported spontaneous healing [11]. Larger defects with diameters of 4 mm to 5 mm may be more appropriate [22, 23]. For the canine model with a mean weight of about 30 kg, the critical size of the OCD has been considered to be 4 mm [19, 24]. Gotterbarm et al. considered that OCD of 6.3 mm should be defined as the critical-sized defect in the porcine model with a mean weight of about 38 kg [25]. The critical-sized defect in sheep models has been considered to be 7 mm, while its average weight is about 70 kg [11]. In the goat model, 6 mm OCD proved to be unable to heal spontaneously and has been defined as a critical dimension defect, while the average weight is about 48 kg [26, 27]. The critical-sized defects in the equine femoral trochlear and condyle models are considered to be around 9 mm [28, 29]. In addition, Salonius et al. [30] reported 4 mm in diameter as critical osteochondral lesion size in the equine carpal joint model. The horse is the largest animal model for articular cartilage regeneration with an average weight of 400 kg.

## 3. Small Animal Models

Small animal models are crucial in “proof-of-concept” studies, especially for testing biosafety. In these studies, concepts are validated and *in vitro* results are first translated *in vivo*. Small animals are inexpensive, easy to handle and feed, and often used to investigate the pathophysiology and pathogenesis of the disease [31]. However, the limitations of small animal models for OCD regeneration consist in the small size of the knee joint and the thin cartilage thickness [32, 33]. It is therefore difficult to design surgical OCD models suitable for comparison with human conditions.

### 3.1. Rats

The rat models used for OCD regeneration have several advantages, as rats are inexpensive, easy to handle and house, and clinically more relevant than mice. The skeletal maturity of rats is approximately 7 months [34]. Rats aged between 9 and 12 weeks have been used to evaluate the degradation rate and safety profile of biomaterials, whereby the experimental period of implants generally lasts 8–12 weeks (Table 2). The critical size of rat OCD was defined as 1.4 mm [20]. The cartilage thickness of the medial femoral condyle in rat is around 0.1 mm [11]. Most commonly, OCD of 2.0 mm diameter and 2.0 mm depth on the trochlear groove of the femur have been used for the assessment of biomaterial strategies. However, their small joint size and thin cartilage remain the main limitations for testing of biomaterials in the rat OCD model [20]. Therefore, the rat model seems to be applicable for preliminary *in vivo* evaluation but not for preclinical studies.

#### 3.1.1. Experimental Protocol of Animal Surgeries

In typical procedures, animals were anaesthetized and shaved and the knee was disinfected. A medial temporal medial longitudinal incision was made to expose the synovium of the knee joint, and then the trochlear groove was further exposed after the lateral patellar luxation. The defect (1.5–2 mm diameter and 2 mm depth) was drilled in the center of the trochlear groove. The biomaterials were implanted, after irrigating the joint with sterile isotonic saline. Lastly, the patella was relocated and the wound sutured in layers [40].

#### 3.1.2. Applications of Rat OCD Model for Testing of Osteochondral Repair Materials

Using a 12-week-old rat model, Lee and Im [35] found that SOX trio-co-transduced adipose tissue derived stem cells (ASCs) in fibrin gel promoted the OCD (2 mm diameter and 2 mm depth) regeneration and attenuated the progression of OA caused by surgery. Muttigi et al. [36] created an OCD of 2 mm diameter and 2 mm depth in the patellar groove of the femur. The model was created to assess the effect of matrilin-3 codelivery with ASCs. They found that matrilin-3 codelivery with ASCs enhanced the formation of cartilage tissue and concluded that matrilin-3 may be an attractive biochemical factor that promotes stem cell repair of articular cartilage. Mahmoud et al. [37] used 10-week-old rat to create an OCD model in the femur patellar groove (2 mm diameter and 2 mm depth) to test the efficacy of multilineage-differentiating stress-enduring (Muse) cell transplantation for OCD repair. They found that injection of Muse cells was a promising method to repair an OCD, especially when subchondral bone is covered by fibrous tissue. Dahlin et al. [38] cultured bovine articular chondrocytes with rat mesenchymal stem cells (MSCs) onto electrospun poly(3-caprolactone) (PCL) scaffolds and implanted them into OCD (2 mm diameter and 2 mm depth) in the rat trochlear groove. The results showed cocultures of articular chondrocytes and MSCs have the potential to repair cartilage defects *in vivo*. Li et al. [39] combined poly(lactide-coglycolide)/hydroxyapatite (PLGA/HA) composite scaffolds with MSCs to successfully repair cartilage defects, while these implants may also be valuable for other clinical applications.

### 3.2. Rabbit

The rabbit model provides a suitable small animal model for assessing the repair of OCD, as rabbits have larger joints for surgical procedures [41]. The age of skeletal maturity in rabbits is 9 months. Rabbits aged between 3 and 8 months have been used to evaluate the degradation rate and safety of biomaterials, and the experimental period of implants generally lasted 8–24 weeks (Table 3). The cartilage of rabbit is relatively thin, showing an average cartilage thickness of 0.44 ± 0.08 mm for the trochlear groove and 0.3 ± 0.07 mm for the medial femoral condyle [47]. In addition, the subchondral bone of the rabbit trochlea (386 ± 160 *μ*m) is similar to the human medial femoral condyle (213 ± 116 *μ*m), and both have a relatively thin bone plate and a more porous and lower density subchondral bone [48]. The relative length of the trochlear groove is greater compared with the human knee joint, which is probably related to the mainly squatting posture of the animal. Besides, the rabbit has faster skeletal change and bone turnover in comparison with other species [49]. Defects have been created in the femoral trochlea [50, 51], the medial femoral condyle [52, 53], and the lateral femoral condyle [54]. OCD of 3.0–5.0 mm diameter and 2.0–5.0 mm depth are often used to evaluate biomaterials in rabbit models.

#### 3.2.1. Experimental Protocol of Animal Surgeries

**Figure 1:**
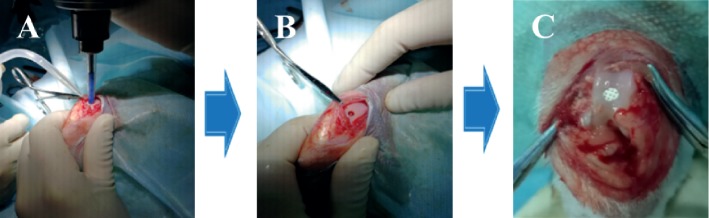
The process of the OCD regeneration in rabbits. A: the OCD were generated by electric drill in the femoral patellar groove; B: a 3.2 mm in diameter and 3.0 mm deep OCD was obtained; C: the biomaterial was implanted into the OCD.

In most studies, the creation of an OCD was based on the following protocol. The rabbits were anaesthetized; then, a medial peripatellar incision was made to expose the knee joint. The patella was dislocated laterally, and the articular surface of the distal femur was exposed. A cylindrical OCD was made using an electrical trephine in the trochlear groove (Figure 1). After irrigating the joint with sterile isotonic saline, the biomaterials were implanted. Lastly, the patella was relocated and the wound sutured in layers [50, 51].

#### 3.2.2. Applications of Rabbit OCD Models for Testing of Osteochondral Repair Materials

Liao et al. [42] prepared a novel hybrid scaffold composed of methacrylated chondroitin sulfate (CSMA), poly(ethylene glycol) methyl ether-*ε*-caprolactone-acryloyl chloride (MPEG-PCL-AC, PECA was used as abbreviation for MPEG-PCL-AC), and graphene oxide (GO) and evaluated its application for cartilage regeneration using the rabbit OCD model. Micro-CT and histological observations showed that the CSMA/PECA/GO scaffold group had better chondrocyte morphology, integration, and continuous subchondral bone and thicker newly formed cartilage. Bauer et al. [43] used a 4 mm diameter and 5 mm depth rabbit OCD model to test hyaluronic acid thioester to promote articular cartilage regeneration. Ruan et al. [44] synthesized a novel biphasic scaffold, which contained a silk-fibroin/chitosan (SF/CS) and an osteoblastic phase (SF/CS/nHA). Bone marrow derived mesenchymal stem cells (BMSCs) showed high cell viability on this scaffold. This scaffold may be an attractive implant that has potential applications in the treatment of OCD. Meng et al. [45] established a functional scaffold named APM-E7 by conjugating a BMSCs affinity peptide (E7) onto the acellular peritoneum matrix (APM). Then, they established a full-thickness OCD model, 4 mm in diameter and 2 mm in height, in 6-month-old rabbits to test the APM-E7 scaffold. The results showed APM-E7 scaffold could support cell attachment. Zhang et al. [46] fabricated a bilayer microporous scaffold with collagen and electrospun poly-L-lactic acid nanofibers (COL-nanofiber) and applied it in a rabbit OCD model. The results showed that implantation of COL-nanofiber scaffold with cells induced cartilage and subchondral bone formation.

## 4. Large Animal Models

The large animals, such as goats, sheep, pigs, dogs, and horses, have the advantages of joint size and cartilage thickness and also have the most similar clinical lesions to humans [55]. Although large animals may be closer to human clinical conditions, they require greater logistic, financial, and ethical considerations. When planning *in vivo* studies, a multivariate analysis should be performed for each animal model. Ultimately, the scientific goals are crucial for determining the appropriate animal model [31]. According to available reports, the mean volume of human cartilage defects is around 552.25 mm^3^, and the diameter of human cartilage defects requiring treatment is usually 10 mm or more [56, 57]. However, in common animal models, the cartilage volume and cartilage thickness are smaller than in humans (Table 2) [11, 58].

### 4.1. Dog

The dog is considered to be a very friendly and loving partner over the world. The social and ethical issues associated with the use of dogs as preclinical and translational animal models are main reasons for their limited use [14]. Dogs are susceptible to cartilage diseases such as exfoliative osteochondritis and osteoarthritis, and dogs lack the ability to repair cartilage defects intrinsically [31]. Therefore, using this model to study osteoarthritis may be closer to humans. Dogs are also suitable for studies that require specific sports and rehabilitation protocols. Dog's skeleton mature age is about 12 to 24 months. The thickness of the cartilage on the medial condyle of the dog has been reported to be 0.95 mm [11]. Defects have been located in the femoral trochlea [59], the medial femoral condyle [60], and both condyles concurrently and medial tibial plateau [61]. Defect diameters have ranged from 2 to 10 mm, and 4 mm is the most common one (Table 4).

#### 4.1.1. Experimental Protocol of Animal Surgeries

Dogs were anaesthetized intravenously. The dog was fixed on the operating table in a supine position and the hair was shaved over the knee joint. The operating field was disinfected, and an incision was created in the skin of the knee joint. The knee flexion was approximately 70°; a defect was created in the femoral trochlea, the medial femoral condyle, or condyles concurrently and medial tibial plateau. Scaffolds were implanted, and the wound layer was sutured [59].

#### 4.1.2. Applications of Dog OCD Models for Testing of Osteochondral Repair Materials

Lv and Yu [59] investigated the articular OCD (6 mm diameter and 12 mm depth) repair using a composite lamellar scaffold of nano-*β*-tricalcium phosphate (*β*-TCP)/collagen (col) I and II with BMSCs in the canine knee joint. The composite lamellar scaffold was gradually degraded and absorbed, while new cartilage tissue was formed. Salkeld et al. [61] used a 6 mm diameter and 11 mm deep OCD in the medial femoral condyle of the canine knee to test a pyrolytic carbon implant. They found that the pyrolytic carbon as a hemiarthroplasty implant material was superior to cobalt-chromium (Co-Cr) alloy. In addition, pyrolytic carbon implants reduced wear, degradation, and cellular changes at the surface of the tibial cartilage. Yamazoe et al. [62] proposed that autologous transplantation of an atelocollagen gel containing canine-derived mesenchymal stem cells could not promote the repair of canine knee joint but rather the subchondral bone regeneration.

### 4.2. Pig

Pigs are considered to be a suitable animal model for mimicking human diseases and have widely been used in biomedical research [63, 64]. The pig joint size, weight requirements, and cartilage thickness are closer to humans than dogs and smaller animal models. In addition, the bone apposition rate and trabecular thickness of the mini-pig are similar to human bones. However, purchase and maintenance of pigs are very expensive. Pigs generally reach skeletal maturity in around 18 months [14]. Fisher et al. [65] reported a cartilage thickness of 1.5 mm at the medial femoral condyle level in mini-pig. Gotterbarm et al. [25] showed that 6.3 mm diameter OCD did not spontaneously heal in mini-pig, confirming the applicability of this pig breed to articular cartilage research. The large majority of the cartilage regeneration studies in the mini-pig are performed on the joint knee, involving the medial [66] or femoral condyles [67, 68], or femoral trochlea. Generally, 6 mm to 8 mm diameter or larger dimensions OCD are created, and the postoperative follow-up period is usually between 3 and 24 months (Table 5).

#### 4.2.1. Experimental Protocol of Animal Surgeries

After animals were anaesthetized, a 5 cm incision was created in the skin to expose the medial condyle. A cylindrical OCD was created in the knee joint. The implant was placed into the defect and taken care of to ensure that the scaffold was flushed with the surface of the surrounding articular cartilage. Lastly, the wound was sutured in layers [66].

#### 4.2.2. Applications of Pig OCD Models for Testing of Osteochondral Repair Materials

Several studies on cartilage and cartilage defects have been reported using min-pig. Christensen and coauthors [68] created OCD of 6 mm diameter and 8 mm depth in the medial trochlear to investigate the role of cartilage chips. They found that the cartilage chips promoted the formation of fibrocartilage rather than fibrous tissue. Betsch et al. [66] found that the combination of erythropoietin (EPO) and bone marrow aspirate concentrate (BMAC) could promote osteochondral healing in mini-pig OCD. Jagodzinski et al. [69] found that stem cell concentrates enhanced the attachment of new bone but did not enhance the mechanical properties and histological appearance of cartilage regenerates in mini-pig OCD models.

### 4.3. Sheep

Sheep is one of the commonly used animal models in orthopaedic research. The anatomy of the knee is similar to humans. However, due to the thinness of the cartilage, most of the defects are located in the subchondral bone, and the skeletal maturation is later, representing certain limitations [11]. Sheep aged between 2 and 3 years have been used to evaluate the degradation rate and safety profile of biomaterials, and the experimental period of implants generally lasted for 16–52 weeks. The critical-sized defect has been reported as 7 mm. The cartilage thickness of the medial femoral condyle is approximately 0.45 mm. The location of the cartilage defects in the sheep model has involved the medial femoral condyle [67, 70, 71], both femoral condyles [72, 73], and the femoral trochlea [70]. OCD with a diameter of 6–8 mm and a depth of 5–13 mm were used for the assessment of biomaterial strategies (Table 6).

#### 4.3.1. Experimental Protocol of Animal Surgeries

The sheep were anaesthetized; then, sheep were placed in dorsal recumbency. The skin on the right knee was sterilized and was ready for sterile surgery. The lateral para-aortic joint was incised to expose the medial and lateral femoral condyles. An ideal OCD was created in the medial and lateral femoral condyles using a suitable drill bit. After irrigating the joint with sterile isotonic saline, the biomaterials were implanted. Lastly, the wound was sutured in layers [72].

#### 4.3.2. Applications of Sheep OCD Models for Testing of Osteochondral Repair Materials

Schlichting et al. [70] created an 8 mm in diameter and 15 mm deep OCD in the femoral condyles of 24 sheep to prove that stiff scaffolds could improve bone and cartilage regeneration. Bernstein et al. [71] indicated that microporous *β*-TCP scaffolds with chondrocytes were favorable for the treatment of OCD using the sheep model. Mohan et al. [72] compared microfracture and osteochondral methods using microsphere-based gradient plugs in sheep models. They found that gradient scaffolds had better cartilage repair capacity for OCD. Yucekul et al. [74] investigated a biodegradable, trilayered poly(glycolic acid) mesh/poly(l-lactic acid)-colorant tidemark layer/collagen type I and ceramic microparticle coated poly(l-lactic acid)-poly(*ε*-caprolactone) monolith) osteochondral plug indicated for the repair of cartilage defects (8 mm × 10 mm) in sheep. The scaffold proved to have a significant positive effect on the healing of osteochondral lesions. Mrosek et al. [75] demonstrated that trabecular metal (TM) was a very suitable material for reconstructing bone defects. TM enabled excellent bone ingrowth and rapid integration.

### 4.4. Goat

Goats are similar to sheep and are easy to raise and manage. The skeletal maturity of goats is similar to that of sheep, namely, about 2 to 3 years [11]. Goats aged between 2 and 4 years have been used to evaluate the degradation rate and safety profile of biomaterials, and the experimental period of implants generally lasted for 6–12 months (Table 7). The thickness of cartilage in goat is greater than that in sheep, and the subchondral bone is softer than that in sheep, which renders goats prone to osteochondral bone defects. Goat joints are usually larger than canine joints, and the most common defect size is 6 mm in diameter; this size has been proven to be unable to heal spontaneously. Defects have been created in the femoral trochlea, the medial femoral condyle, the lateral femoral condyle, and the talus [76, 77, 81]. If the limitations of large animal models can be overcome, including higher costs and adequate facility requirements, the goat model is a viable large animal model for cartilage and osteochondral lesions. However, the size of the lesions is still smaller than the human-related clinical diagnosis (Table 7).

#### 4.4.1. Experimental Protocol of Animal Surgeries

Surgery was performed under general anesthesia via joint surgery. Using retractors with the limb placed at maximal flexion, the implantation site was exposed. Defect was created and an implant was inserted via a surgical tool. The implant reached its final position in a press-fit manner, slightly below the articular surface. The knee capsule and skin were then sutured.

#### 4.4.2. Applications of Goat OCD Models for Testing of Osteochondral Repair Materials

Goat has been successfully used as a model for OCD to evaluate new implants. Zhang et al. [76] fabricated BMSC-integrated osteochondral scaffolds that could promote the repair of OCD in goats. van Bergen et al. [77] used a 6 mm OCD in the talus goat model to evaluate the effectiveness of demineralized bone matrix (DBM) with and without platelet-rich plasma (PRP). They found that PRP would further enhance the regenerative capacity of DBM. Kon et al. [78] created critical-sized defects of 6 mm diameter and 10 mm depth in the medial femoral condyle of the knee joint. The defect model was created to test the *in vivo* effect of aragonite-hyaluronate (Ar-HA) scaffolds. They found that the Ar-HA scaffold might induce cartilage and subchondral bone regeneration. Sun et al. [79] evaluated the efficacy of gene enhanced tissue engineering following mosaicplasty in a goat model. They found that gene enhancement could effectively restore a 9 mm diameter OCD in a goat model. Pei et al. [80] used the goat OCD model and implanted a tissue-engineered osteochondral (TEO) graft to investigate its reparative efficacy. Their results showed that this TEO was a promising substitute biomaterial for osteochondral regeneration.

### 4.5. Horse

As horses are robust and long-lived animals, they are suitable models for assessing the repair of superficial cartilage and subchondral bone in chronic injuries in weight-bearing conditions. Similar to humans, the horses suffer from cartilage diseases and have very weak cartilage self-repairing ability [82]. It is reported that the thickness of articular cartilage is 1.75 mm, which is closest to human cartilage thickness (2.35 mm). Cartilage and OCD of 15 to 20 mm can be assessed in horses. In addition, the upright knee joint with large joint size, thick joint cartilage, and fully straightened gait process is closer to the human knee anatomy than the other animal models. The age of skeletal maturity in the horse is 2–4 years. The age of horses used ranges from 2 to 6 years. Defects have been created in the femoral trochlea [83], the medial femoral condyle [84], the lateral trochlear ridge [85], and the medial surface of lateral trochlea of the talus [86]. A 10 mm in diameter and 5 mm–10 mm deep defect has often been created to simulate osteochondral defects. The major disadvantages of equine models include high cost, inconvenient management, and long-term care during and after surgery. High joint load conditions, high prices, and the need for highly specialized facilities limit the use of horse models for researchers (Table 8).

#### 4.5.1. Experimental Protocol of Animal Surgeries

Horse was positioned in dorsal recumbence. General anesthesia was maintained and a 5 cm incision made between the middle and medial patellar ligaments. OCD were created using a power-driven drill. Defect site and joints were flushed with saline solution before implantation. Scaffolds were press-fit implanted into each defect. Wounds were sutured in four layers (joint capsule, deep fascia, superficial fascia, and skin) and a stent bandage was applied over the incision [84].

#### 4.5.2. Applications of Horse OCD Models for Testing of Osteochondral Repair Materials

Bolanos et al. [84] used a horse model to investigate the effect of decellularized cartilage-derived matrix (CDM) scaffolds with a calcium phosphate (CaP) base for the repair of OCD. Seo et al. [83] evaluated the efficacy of a synovial flap and gelatin/*β*-tricalcium phosphate (GT) sponge loaded with mesenchymal stem cells (MSCs), bone morphogenetic protein-2 (BMP-2), and platelet-rich plasma (PRP) for repairing of OCD in horses. The results showed that the GT/MSCs/BMP-2/PRP implantation promoted osteochondral regeneration in the equine model. McCarrel et al. [85] used a 10 mm in diameter and 10 mm deep equine model to test a biphasic cartilage repair device (CRD) for feasibility of arthroscopic implantation and long-term repair of OCD. Maninchedda et al. [86] established a 10 mm in diameter and 5 mm deep OCD model in 3-year-old horses, and the defect was filled with chitosan-GP. After 180 days, they found that the implanted chitosan-GP did not cause any important inflammatory reaction and allowed cell growth.

## 5. Nonhuman Primate Model

Most animal models differ in biomechanical functions and/or physiological responses from human, limiting the ability to extrapolate data to clinical practice. The nonhuman primate (NHP) models overcomes many of these limitations, as they have similar genetic, physiological, and behavioral characteristics to humans and can highly mimic human health issues [87, 88]. Some reports have used NHP to study cartilage regeneration. Kagimoto et al. used a monkey model to assess the safety and efficacy of the xenotransplantation of human cartilage progenitor cells. They found that autologous transplantation of cartilage progenitor cells may be effective in repairing elastic cartilage [89]. Buckwalter et al. used skeletally mature cynomolgus monkeys to create 3.2 mm in diameter and 4.0 mm deep osteochondral defects of the articular surfaces of the patella (PA) and the medial femoral condyle (FC) in both knees and then treated them with intermittent passive motion (IPM) or cast-immobilization (CI). However, they found that repair of acute osteochondral damage in primates failed to restore normal articular surfaces within eight weeks [90]. Ma et al. suggested that the chondrogenic clonal MSC-loaded monkey acellular dermal matrix (MSC-ADM) scaffold can improve cartilage damage in cynomolgus monkey models and can be used to repair similar human cartilage defects [91]. Jiang et al. made 3 mm in diameter and 2 mm deep cartilage defects on the distal femurs surface of cynomolgus monkeys and treated them with autologous selected chondrogenic clonal MSCs (sC-MSCs). They found that sC-MSCs can effectively improve the healing of cartilage damage in monkey OA induced by collagenase [92] (Table 9). Despite having big similarity to humans, NHP have been seldom utilized in cartilage regeneration research, due to scarcity, high costs, ethical consideration, and high profile in animal welfare and also because these are often unable to provide additional information beyond the aforementioned large animal models.

## 6. Selecting an Appropriate Animal Model Based on Multiple Factors

The selection of animal models is critical to promote translational research to the clinical application of biomaterials. Generally, small animal models including rats and rabbits are beneficial for early-phase testing, such as testing degradation, biocompatibility, and interaction of implanted biomaterials with host tissues. Because they are economical and easy to handle and have short time for healing (usually 12 weeks for rabbits) [19], large animals are more suitable for late-phase translational research because their articular cartilage structure is much similar to the mechanical load on humans [93, 94]. However, large animal study is often limited by high costs, long duration (at least 24 weeks), or even ethics. For example, it is difficult to obtain ethical permission to use dogs in some countries or districts pertaining to their companion animal status. Multiple factors should be considered for selecting the appropriate animal models to achieve specific study objectives, such as the size and location of the defect, age, study duration, and surgical considerations. Besides scientific evaluation, the choice is also influenced by practical aspects such as ethics, costs, and housing.

## 7. Conclusion

In this review, we summarize the benefits and limitations of each species for reproducing specific defects, analyze and compare the similarities between animal models and human clinical situations, and emphasize the factors we need to consider when choosing animals. This review provides an important reference for selecting a suitable animal model(s) for the development of new strategies for osteochondral regeneration.

## Figures and Tables

**Table 1:** Comparison of age, cartilage, and defect size in different species.

Species	Age of skeletal maturity	Cartilage thickness	Cartilage volume	Critical-sized defect	Common defect depth
Rat	7 months	0.1 mm	2.17 mm^3^	1.4 mm	1.0–2.0 mm
Rabbit	9 months	0.3 mm	53 mm^3^	3.0 mm	3.0–5.0 mm
Dog	12‐24 months	0.95 mm	82.39 mm^3^	4.0 mm	10–12 mm
Pig	18 months	1.5 mm	107.47 mm^3^	6.3 mm	8–10 mm
Sheep	2‐3 years	0.45 mm	359.54 mm^3^	7.0 mm	6–13 mm
Goat	2‐3 years	1.1 mm	251.65 mm^3^	6.0 mm	6–12 mm
Horse	2‐4 years	1.75 mm	334.73 mm^3^	4.0 mm/9.0 mm	10 mm
Monkey	10 years [16]	0.5–0.7 mm [17]	—	—	2–4 mm
Human	18–22 years	2.35 mm	552.25 mm^3^	—	—

**Table 2:** Examples of studies using rat osteochondral defect models.

Authors	Age	Defect size (diameter × depth)	Location	Endpoint	Material tested
Lee and Im [35]	12 weeks	2 mm × 2 mm	The trochlear groove of the femur	8 weeks	SOX trio-co-transduced ASCs
Muttigi et al. [36]	12 weeks	2 mm × 2 mm	The center of the groove	12 weeks	Matrilin-3/mesenchymal stem cell
Mahmoud et al. [37]	10 weeks	2 mm × 2 mm	The patellar groove of the femur	4, 12 weeks	Muse cells
Dahlin et al. [38]	10–12 weeks	2 mm × 2 mm	The center of the trochlear groove	4, 8 weeks	PCL scaffold/MSC
Li et al. [39]	12 weeks	1.5 mm × 2 mm	The trochlear groove	6, 12 weeks	PLGA/HA-MSC

**Table 3:** Examples of studies using rabbit osteochondral defect models.

Authors	Age/weight	Defect size (diameter × depth)	Location	Endpoint	Material tested
Liao et al. [42]	2–2.5 kg	4 mm × 3 mm	The trochlear groove	6, 12, and 18 weeks	CSMA/PECA/GO hybrid scaffold
Bauer et al. [43]	8 months	4 mm × 5 mm	The medial trochlear groove	4 and 12 weeks	Hyaluronic acid thioester
Ruan et al. [44]	6 months	4 mm × 3 mm	The medial trochlear groove	4, 8, and 12 weeks	SF/CS/nHA phase scaffold
Meng et al. [45]	4–6 months	4 mm × 2 mm	The trochlear groove	6, 12, and 24 weeks	AMP-E7/BM-MSC
Zhang et al. [46]	2.5–3 kg	4 mm × 4 mm	The patellar groove	6 and 12 weeks	COL-nanofiber and COL scaffolds

**Table 4:** Examples of studies using dog osteochondral defect models.

Authors	Age	Defect size (diameter × depth)	Location	Endpoint	Material tested
Lv and Yu [59]	12 months	6 mm × 12 mm	The right knee joint	12 and 24 weeks	Nano-*β*-TCP/Col I/Col II/BMSCs
McCarty et al. [60]	—	4.5 mm × 10 mm	The medial femoral condyle	12 months	Osteochondral allograft
Salkeld et al. [61]	1.6 years	6 mm × 11 mm	The medial femoral condyle and medial tibial plateau surfaces	12, 24, and 52 weeks	Pyrolytic carbon scaffold and Co-Cr alloy scaffold
Yamazoe et al. [62]	1–3 years	5 mm × 4.5 mm	The femoral condyles	2, 4, and 10 weeks	Atelocollagen gel/MSCs

**Table 5:** Examples of studies using pig osteochondral defect models.

Authors	Age	Defect size (diameter × depth)	Location	Endpoint	Material tested
Christensen et al. [68]	19.8 months	6 mm × 8 mm	The medial trochlear and the lateral trochlear	6, 24 months	Autologous dual-tissue transplantation/autologous cartilage chips
Betsch et al. [66]	18–30 months	6 mm × 10 mm	The medial femoral condyle	26 weeks	EPO/BMAC/scaffold
Jagodzinski et al. [69]	14 months	7 mm × 10 mm	The medial or lateral femoral condyles	3 months	Bone marrow derived cell concentrates

**Table 6:** Examples of studies using sheep osteochondral defect models.

Authors	Age	Defect size (diameter × depth)	Location	Endpoint	Material tested
Schlichting et al. [70]	2 and 3 years	7.3 mm × 10 mm	The femoral condyles	3, 6 months	Stiff scaffold
Bernstein et al. [71]	2–4 year	7 mm × 25 mm	The femoral condyles	6, 12, 26, and 52 weeks	*β*-TCP/chondrocytes
Mohan et al. [72]	>3.5 years	6 mm × 6 mm	MFCs and LFCs	1 year	PLGA/*β*-TCP
Yucekul et al. [74]	—	8 mm × 10 mm	The lateral condyles	3, 6 and 12 months	PLLA/PCL/*β*-TCP
Mrosek et al. [75]	—	8 mm × 13 mm	The medial femoral condyle	16 weeks	Trabecular metal with an autologous periosteum graft

**Table 7:** Examples of studies using goat osteochondral defect models.

Authors	Age/weight	Defect size (diameter × depth)	Location	Endpoint	Material tested
Zhang et al. [76]	12 months	6 mm × 8 mm	Knee joint	12, 24 weeks	BMSC-integrated osteochondral scaffolds
van Bergen et al. [77]	4-year-old	6 mm × 6 mm	Knee joint	24 weeks	Demineralized bone matrix
Kon et al. [78]	2-year-old	6 mm × 10 mm	The load-bearing medial femoral condyle	24 weeks	Aragonite-hyaluronate
Sun et al. [79]	22.5 kg	9 mm × 3 mm	The weight bearing area of the medial femoral condyle	24 weeks	Gene enhanced tissue engineering followed mosaicplasty
Pei et al. [80]	—	6 mm × 12 mm	The femoral medial condyle weight-bearing area	12 and 24 weeks	Tissue-engineered osteochondral graft

**Table 8:** Examples of studies using horse osteochondral defect models.

Authors	Age	Defect size (diameter × depth)	Location	Endpoint	Material tested
Seo et al. [83]	3.6 ± 2.3 years	10 mm × 5 mm	The medial condyle	6 months	GT/MSCs/BMP-2/PRP implantation
Bolanos et al. [84]	6 years	11 mm × 10 mm	The middle aspect of medial femoral trochlear ridge	6 months	CDM/CaP
McCarrel et al. [85]	2–5 years	10 mm × 10 mm	The lateral trochlear ridge	4, 12, and 24 months	Biphasic cartilage repair device
Maninchedda et al. [86]	3 years	10 mm × 5 mm	The medial surface of lateral trochlea of talus	6 months	Type II collagen

**Table 9:** Examples of studies using monkey cartilage or osteochondral defect models.

Authors	Age	Defect size (diameter × depth)	Location	Endpoint	Treatment
Buckwalter et al. [90]	—	3.2 mm × 4 mm	The patella and the medial femoral condyle	8 weeks	Intermittent passive motion (IPM) or cast-immobilization (CI)
Ma et al. [91]	3–5 years old	3.2 mm × 2 mm	Knee joints	24 weeks	MSC-loaded ADM scaffold
Jiang et al. [92]	3–5 years old	3 mm × 2 mm	The surface of distal femurs	24 weeks	Autologous selected chondrogenic clonal MSCs
